# Spatiotemporal Dynamics of Potential Distribution Patterns of *Nitraria tangutorum* Bobr. Under Climate Change and Anthropogenic Disturbances

**DOI:** 10.3390/plants14172706

**Published:** 2025-08-30

**Authors:** Yutao Weng, Jun Cao, Hao Fang, Binjian Feng, Liming Zhu, Xueyi Chu, Yajing Lu, Chunxia Han, Lu Lu, Jingbo Zhang, Tielong Cheng

**Affiliations:** 1 College of Ecology and Environment, Nanjing Forestry University, Nanjing 210037, China; wengyutao99@163.com (Y.W.); jasonfangplus1609@njfu.edu.cn (H.F.); chuchuchuyi@163.com (X.C.); lanieyajing2022@163.com (Y.L.); 2 State Key Laboratory of Tree Genetics and Breeding, Co-Innovation Center for Sustainable Forestry in Southern China, Nanjing Forestry University, Nanjing 210037, China; caojun2025@163.com (J.C.); fbj@njfu.edu.cn (B.F.); zhulm@njfu.edu.cn (L.Z.); lulu2020@njfu.edu.cn (L.L.); 3 Experimental Center of Desert Forestry, Chinese Academy of Forestry, Dengkou 015200, China; hanchunxia0116@126.com (C.H.); nmzhangjb@126.com (J.Z.)

**Keywords:** *Nitraria tangutorum*, MaxEnt, human footprint, future climate scenarios, MESS

## Abstract

Under the context of global climate change, the frequent occurrence of extreme low-temperature events poses a severe challenge to plant distribution and ecosystem stability. The arid and semi-arid regions of Northwestern China, as a sensitive response area to global change, have proven to possess significant development potential with their unique desert vegetation systems. This study focuses on the ecological adaptability mechanisms of *Nitraria tangutorum* Bobr., a key species of the desert ecosystem in Northwestern China, and systematically analyzes the evolution patterns of its geographical distribution under the coupled effects of climate change and human activities through a MaxEnt model. The research conclusions are as follows: (i) This study constructs a Human Footprint-MaxEnt (HF-MaxEnt) coupling model. After incorporating human footprint variables, the AUC value of the model increases to 0.914 (from 0.888), demonstrating higher accuracy and reliability. (ii) After incorporating human footprint variables, the predicted area of the model decreases from 2,248,000 km^2^ to 1,976,000 km^2^, with the High Suitability experiencing a particularly sharp decline of up to 79.4%, highlighting the significant negative impact of human disturbance on *Nitraria tangutorum*. (iii) Under the current climate baseline period, solar radiation, precipitation during the wettest season, and mean temperature of the coldest month are the core driving factors for suitable areas of *Nitraria tangutorum*. (iv) Under future climate scenarios, the potential distribution area of *Nitraria tangutorum* is significantly positively correlated with carbon emission levels. Under the SSP370 and SSP585 emission pathways, the area of potential distribution reaches 172.24% and 161.3% of that in the current climate baseline period. (v) Under future climate scenarios, the distribution center of potential suitable areas for *Nitraria tangutorum* shows a dual migration characteristic of “west–south” and “high altitude”, and the mean temperature of the hottest month will become the core constraint factor in the future. This study provides theoretical support and data backing for the delineation of habitat protection areas, population restoration, resource management, and future development prospects for *Nitraria tangutorum*.

## 1. Introduction

The Northwest Desert Area, as a highly fragile typical region within China’s ecological security framework, faces extreme arid climate conditions (annual precipitation <150 mm), severe hydrothermal fluctuations (annual temperature range >35 °C, diurnal temperature range >20 °C), and a degraded hydrological system, forming critical bottlenecks for regional ecological stability [1]. Under the synergistic effects of global climate change and human disturbances, the ecosystem services in this region continue to deteriorate. Climate extremization is intensifying, compounded by human-induced overgrazing, reduced water resources, and deteriorated water quality, ultimately leading to the decline in desert plant diversity and limited availability of resources for ecological restoration [2,3]. Studies have shown that climate change significantly alters the spatial distribution patterns of desert plants through temperature threshold effects [4]. As a climate-sensitive response area, the mechanisms of ecological adaptability of key species within the Northwest Desert Area have become a research focus. *Nitraria tangutorum*, as a typical desert constructive species, exhibits multiple ecological adaptability traits such as drought resistance, salt tolerance, and low-temperature resilience [5,6,7]. Its unique ecological plasticity provides an important theoretical paradigm for desert vegetation restoration [8].

*Nitraria tangutorum* commonly known as Tangut Nitraria or desert cherry, is a clump-forming and deep-rooted perennial shrub in the Nitrariaceae [9,10,11] (https://db.kib.ac.cn/CNFlora/SearchResult.aspx?CPNI=CPNI-024-07016, http://www.efloras.org/florataxon.aspx?flora_id=2&taxon_id=242334052, accessed on 11 August 2025). This species is native to China and Mongolia, and its distribution is quite extensive, primarily in the central and western Inner Mongolia, northern Shaanxi, Hexi Corridor in Gansu, Ningxia Autonomous Region, Xinjiang, Qinghai, and eastern Tibet [12,13,14,15,16,17,18,19]. This species has adapted to withstand drought, cold, wind, and soil salinity conditions [6,20]. The sand dunes formed (known as “Nebkha”) [21,22,23] by its accumulation can effectively stabilize drifting sand and reduce the ecological threats posed by wind and sand in the local area. The shrub has a prostrate growth form, reaching 1–2 m in height, and the root system can grow up to over fifteen meters in length [24,25]. It has succulent leaves, and produces edible berries that can play a unique role in improving the digestive and circulatory systems [26,27,28].

Species Distribution Models (SDMs) integrate species distribution data with environmental variables to build frameworks for predicting potential distribution ranges and habitat suitability [29,30]. The MaxEnt model establishes a nonlinear relationship between environmental variables and species occurrence probability by solving the entropy maximization problem with constraints, allowing for multi-scenario dynamic prediction models [31]. It has advantages, including stable quantitative assessment [32], independence from reliance on non-existence species points [33], and dynamic reflection of environmental factor-driven mechanisms [34]. Duan et al. [6] and Lu et al. [35] predicted the potential distribution of the *Nitraria* at the national-scale (China) and global-scale, respectively, with excellent simulation results. Halimujiang simulated the distribution of four *Nitraria* species, thereby filling the gap in research on the single-species potential distribution of *Nitraria* [36]. However, the above-mentioned research findings still have certain limitations, such as sensitivity insufficiency to sparse distribution data [37] and a lack of representation for human factors. In response to these limitations, we developed a coupled climate–human activity-driven species distribution model grounded in the maximum entropy principle. It integrates multi-source environmental variables (climate, topography, soil, and human activity intensity index), filters dominant environmental factors via the Jackknife method, and validates model accuracy through receiver operating characteristic (ROC) curve analysis. Additionally, we introduce Multivariate Environmental Similarity Surfaces (MESS) and Least Similar Variable Analysis (LSVA) to enhance predictive precision under future climate scenarios, thereby enhancing the interpretation of climate change impacts on the potential distribution of *Nitraria tangutorum*.

This study quantifies the driving effects of climate change and human disturbance on the geographic distribution of *Nitraria tangutorum* through coupling Species Distribution Models (SDMs) with environmental factor-driven mechanisms, revealing the ecological resilience and adaptive evolutionary potential of this species, aiming to provide more actionable bases for decision-making in the construction of ecological security barriers in arid regions.

## 2. Materials and Methods

### 2.1. Study Area Overview

*Nitraria tangutorum*, as a typical constructive species in desert regions, exhibits a natural distribution strongly correlated with the ecological patterns of arid zones in Northwestern China. This study defines the geographical scope as regions with annual precipitation below 200 mm and an aridity index above 4 [38]. Such areas exhibit distinct hydrological characteristics—e.g., scarce surface runoff, predominantly endorheic river systems, arid soils, and vegetation structured around typical desert community structures. Specifically, the study region encompasses the Junggar Basin, Tarim Basin, and Eastern Xinjiang Basin in Xinjiang, and extends to the Hexi Corridor in Gansu, the Qaidam Basin in Qinghai, and the Alxa Plateau in western Inner Mongolia, comprehensively covering the core distribution zone of Northwestern China’s desert ecosystem.

### 2.2. Distribution Data

**Figure 1:**
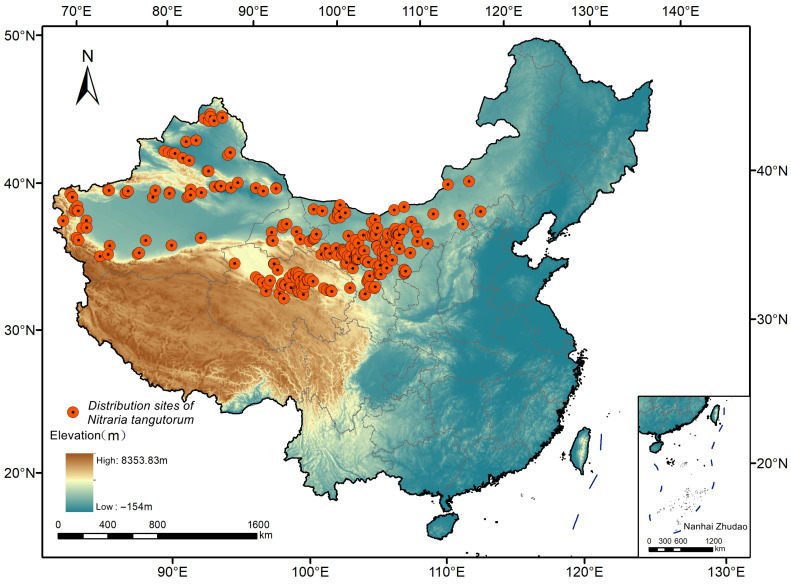
The distribution map of *Nitraria tangutorum* in China.

The research team conducted field surveys in multiple provincial administrative regions, including Xinjiang, Qinghai, Gansu, Inner Mongolia, Ningxia, and Shaanxi, during May–August from 2022 to 2024, collecting data from 61 distribution sample points of *Nitraria tangutorum*. Additionally, 466 species distribution records were sourced from the Global Biodiversity Information Facility (GBIF, 234 records, https://doi.org/10.15468/dl.mvyu48, accessed on 26 July 2024), the Chinese Virtual Herbarium (CVH, 51 records, https://www.cvh.ac.cn/, accessed on 26 July 2024), the National Specimen Information Infrastructure (NSII, 102 records, http://www.nsii.org.cn/2017/, accessed on 26 July 2024), and relevant literature (79 records). After removing duplicates, a total of 392 species distribution records remained. To mitigate overfitting caused by excessive samplings, we apply the Trim duplicate occurrences function in ENMTools v1.3 to filter spatially redundant records within the same 1 km × 1 km grid cell [39], retaining only one occurrence per grid. Non-natural distribution records and duplicate points were excluded, resulting in 260 valid distribution points. Figure 1 depicts the spatial distribution of these filtered points across China.

### 2.3. Environmental Factors

We employed 115 environmental variables for current scenario predictions, encompassing climate, soil, topography, and socioeconomic factors. All data were spatially resolved at 30 arc-s (1 km × 1 km). Climate variables (1970–2000, 2041–2060, and 2081–2100 (future climate scenario periods)), monthly climate data (mean, maximum, and minimum temperature; radiation; wind speed), and bioclimatic data were sourced from the WorldClim database (https://www.worldclim.org/, accessed on 4 September 2024). For future climate scenarios, we adopted the BCC-CSM2-MR model from CMIP6, aligned with China’s geographic context, including three shared socioeconomic pathways (SSPs):

- SSP1-2.6 (sustainable development, ≤2 °C warming by 2100);
- SSP3-7.0 (regional rivalry, ≤4.1 °C warming);
- SSP5-8.5 (fossil-fueled development, ≤5 °C warming) [40].

Evapotranspiration data originated from the National Tibetan Plateau Data Center (TPDC, https://data.tpdc.ac.cn/home, accessed on 15 September 2024). Soil data were sourced from the official FAO of the United Nations Food and Agriculture Organization (FAO, https://www.un.org/zh/node/55053, accessed on 4 September 2024). Topographic data were from the ETOPO Global Relief Model, extracted using ArcGIS software surface analysis tools from digital elevation data. Human footprint data (Human Footprint) were provided by the MuHaowei team [41], which derive human footprint variables through the integration of factors such as population density, nighttime lights, built environment, roads, railways, navigable waterways, and farmlands. These data are published in *Scientific Data*, based on the latest available data from 2000 to 2018, and have been widely cited and referenced, possessing high predictive reliability.

### 2.4. Model Construction and Evaluation

#### 2.4.1. Environmental Factor Screening

**Figure 2:**
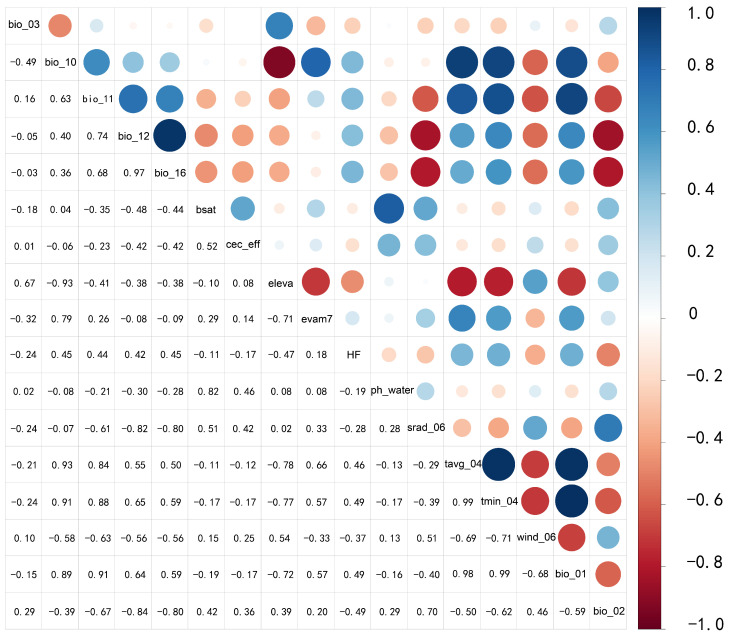
Screening correlation plots of environmental factors under the current climate baseline period.

**Figure 3:**
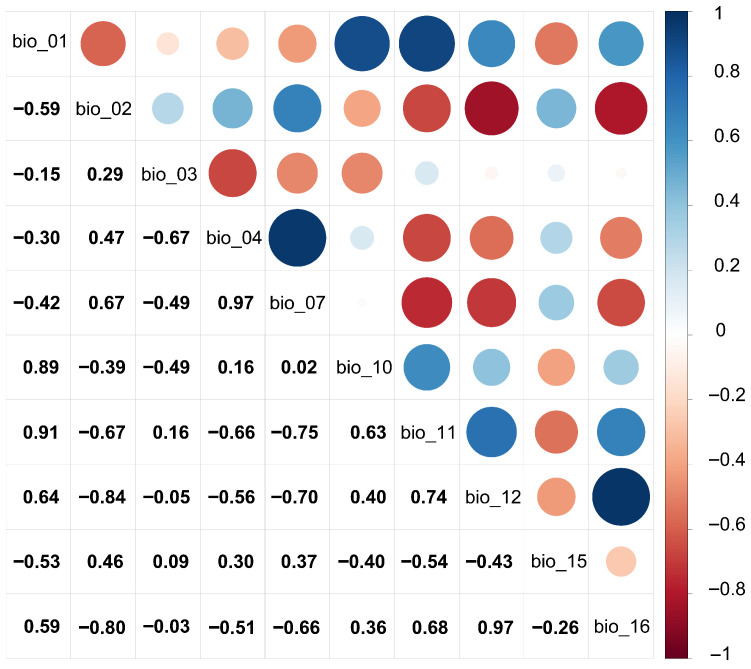
Correlation analysis of environmental factors in future climate scenarios.

When constructing species distribution models, an excessive number of variables can lead to pronounced multicollinearity among them, resulting in the model overfitting the training dataset [42,43,44]. To address this, we initially screened 115 environmental variables using ENMTools.pl (Perl) software 5.26 to perform Pearson correlation analysis. Variables with absolute correlation coefficients of |r| ≥ 0.8 were retained, while those with negligible ecological relevance to *Nitraria tangutorum* were discarded, yielding the screened variable list presented in Table 1. A heatmap of the correlation among screened factors was created using R 4.4.2 software as shown in Figure 2. For 19 variables under future climate scenarios, we adopted a similar optimization strategy: ecologically insignificant variables for *Nitraria tangutorum* were removed, and 10 key factors were ultimately retained based on their dominant roles in species–environment relationships. Table 2 presents the future climate scenarios’ environmental factor data screened in this study, and a heatmap of their correlations was similarly created using R 4.4.2 software as shown in Figure 3. Topographic factors and soil properties, as environmental parameters with spatiotemporal stability, exhibit quasi-static characteristics within the time scale of the study and thus maintaining consistent spatial heterogeneity across the research period. Other environmental variables, due to a insufficient predictive data availability for future climate scenarios (e.g., socioeconomic drivers, frequency of extreme climatic events), were not included in the species distribution simulation driven by future climate scenarios.

#### 2.4.2. MaxEnt Model Settings

We employed Maxent v3.4.4 for model construction. The regularization multiplier (RM) and feature combination (FC) were calibrated using the kuenm package in R [45], with the optimal model selected based on the smallest ΔAICc value following the Akaike Information Criterion [46]. The dataset was partitioned into a 25% test dataset and a 75% training dataset. Model runs were repeated 10 times using a non-parametric subsampling scheme, with outputs formatted as Logistic. Model performance was evaluated using the area under the receiver operating characteristic curve (AUC). The AUC values were interpreted as follows:

- 0.9–1.0: Excellent predictive performance;
- 0.8–0.9: Good predictive performance;
- 0.7–0.8: Moderate predictive performance;
- 0.6–0.7: Poor predictive performance;
- 0.5–0.6: Failed predictive performance [47].

#### 2.4.3. Potential Suitable Habitat Classification

After model construction, MaxEnt output was imported into ArcGIS 10.8 software and overlaid with base maps to generate the potential suitable habitat distribution map for *Nitraria tangutorum*. For the hierarchical classification methodology, a dual-track framework is adopted, consisting of two components: (1) scientific demarcation using the Maximum Test Sensitivity and Specificity (MTSPS) threshold method [48], which precisely calculates habitat suitability indices (HSIs) via the R *kuenm* package; and (2) expert analysis incorporating ecological significance, leveraging the four-tier grading system proposed by Duan Yizhong et al. [6] and adapted from ecological adaptation studies of *Nitraria* spp. [36]. In the operational workflow, core suitable habitats (where HSI ≥ MTSPS threshold) were first delineated, with remaining areas designated as unstable areas. Based on regional climate gradients, a three-tier suitability classification was finalized:

- Low suitability (MTSPS value ≤ *p* < 0.4);
- Moderate suitability (MZ) (0.4 ≤ *p* < 0.6);
- High suitability (HZ) (suitability ≥0.6).

#### 2.4.4. Multivariate Environmental Similarity Surfaces (MESS) and Least Similar Variables Analysis

Considering the impact of the limited availability of existing distribution data for *Nitraria tangutorum* on the reliability of model predictions, relying solely on the MaxEnt model for large-scale spatial projection is prone to significant errors and uncertainties [49]. This study integrates the Multivariate Environmental Similarity Surfaces (MESS) and Most Dissimilar Variable Analysis (MOD) methods [50] to reduce model bias caused by data deficiency. Using the built-in “*density.tools.Novel*” module in MaxEnt, this study constructs a comparative framework between the current climate baseline period (1970–2000) and future climate scenarios (2040–2060, 2080–2100), covering six future climate scenarios (SSP1-2.6, SSP3-7.0, SSP5-8.5, etc.), to analyze potential changes in habitat suitability under different pathways. By identifying strictly extrapolated zones through the MESS index-derived thresholds, the predictive reliability of model predictions is quantified; additionally, the dominant most dissimilar variables are extracted to reveal key driving factors in climate anomaly regions.

#### 2.4.5. Optimal Planting Zone Delineation

This study employs the spatiotemporal overlay analysis method to construct a priority planting zoning model for *Nitraria tangutorum*. Firstly, by integrating multi-temporal suitability data and using the spatial analysis tools of the ArcGIS10.8 platform, fuzzy overlay calculations are conducted to generate a Stable Suitability Zone [51]. Areas with a suitability index above 0.5 are marked using ArcGIS and designated as an optimal planting zone.

## 3. Results

### 3.1. Model Optimization and Accuracy Evaluation

**Figure 4:**
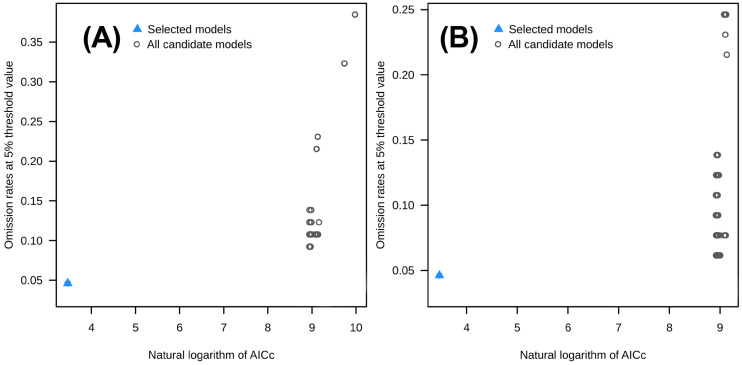
Evaluation metrics of MaxEnt model generated by ENMeval with and without human interference. (**A**,**B**) Scatter plots of the data omission rate and AICc values for models with missing human disturbance and with existing human disturbance.

**Figure 5:**
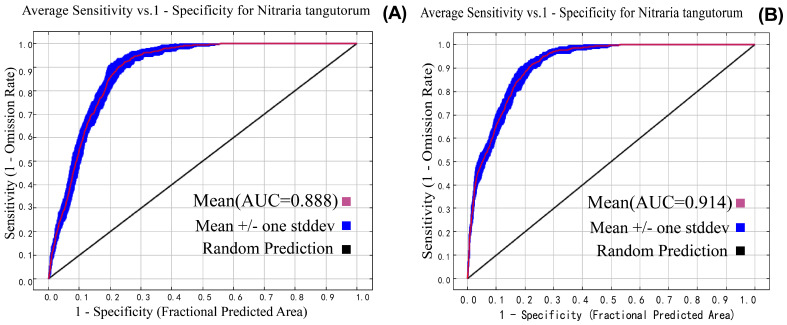
ROC curve of *Nitraria tangutorum* with and without human interference. (**A**,**B**) The model results of area under curve with missing human disturbance and with existing human disturbance.

This study calibrated the selection of environmental variables, with or without human disturbance, using the kuenm package, yielding the following optimal parameters: regularization multiplier (RM), 0.2; and the best feature combination (FC), L (Figure 4). Based on the optimal model configuration, this study conducted 10 independent simulation runs for two scenarios: without human disturbance and with human disturbance. The results showed that the average AUC value for 10 simulations under the scenario without human disturbance was 0.888, while the average AUC value for the scenario with human disturbance was 0.914. The AUC values of the models in both scenarios indicate that the predictions of the models exhibit high accuracy and reliability. Model AUC results are shown in Figure 5.

We further calibrated the environmental variables involved in future climate scenarios, obtaining the following optimal parameters: regularization multiplier (RM), 0.2; and the best feature combination (FC), L. Based on the optimal model configuration, this study conducted 10 independent simulation runs coupling the current climate baseline period with three SSP shared socioeconomic pathways for the periods (2041–2060 and 2081–2100), obtaining an average AUC value of 0.887 across the 10 simulations, indicating good model prediction performance.

### 3.2. The Suitable Habitat Distribution of Nitraria tangutorum in Modern Times

**Figure 6:**
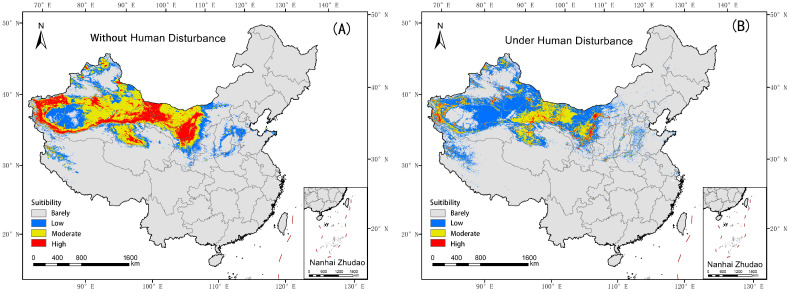
The potential suitable habitat distribution and changes in *Nitraria tangutorum* (**A**) Suitable habitat of *Nitraria tangutorum* without human footprint. (**B**) Suitable habitat of *Nitraria tangutorum* with human footprint.

Under conditions without human disturbance (HD), the potential suitable habitat of *Nitraria tangutorum* is primarily located in China’s Inner Mongolia Autonomous Region, Xinjiang Uygur Autonomous Region, Gansu Province, Qinghai Province, and Ningxia Autonomous Region (Figure 6A). The Highly Suitable area is primarily distributed in the vicinity of the margins of the Tarim Basin, the Zhongshan Hills of Mazong Mountain, the Hetao Plain, and the Helan Mountain region, covering an area of approximately 503,700 km^2^ (22.40% of the total suitable habitat). The Moderately Suitable area spans about 990,100 km^2^ (44.04%), while the Lowly Suitable area occupies 754,400 km^2^ (33.56%) (Table 3).

**Figure 7:**
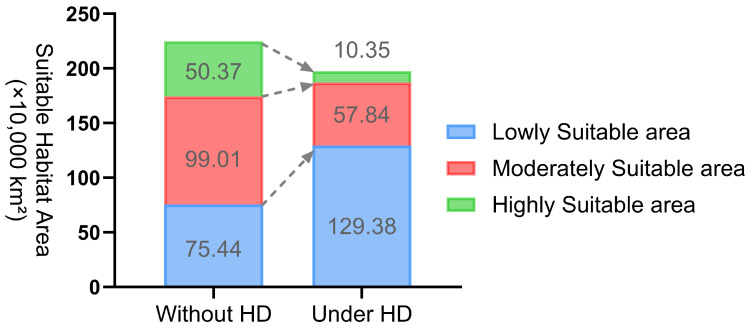
The distribution area of *Nitraria tangutorum* with and without human disturbance. Without HD and Under HD represent without human disturbance and under human disturbance in sequence.

Human disturbance has significantly negatively impacted the distribution of *Nitraria tangutorum*, with its potential suitable habitat appearing highly fragmented and patchy (Figure 6B). In the major distribution administrative regions such as Inner Mongolia Autonomous Region, Xinjiang Uyghur Autonomous Region, Gansu Province, Qinghai Province, and Ningxia Autonomous Region, the potential suitable habitat for *Nitraria tangutorum* has undergone significant large-scale degradation and shrinkage. The Highly Suitable area now accounts for only 103,500 km^2^ (5.24% of the total suitable habitat). The Moderately Suitable area has been reduced to 578,400 km^2^ (29.27% of the suitable habitat). Conversely, the Moderately Suitable area has increased to 1,293,800 km^2^, representing 65.48% of the total suitable habitat (Table 3, Figure 7).

### 3.3. Environmental Factors Influencing the Potential Suitable Habitat Distribution of Nitraria tangutorum

**Figure 8:**
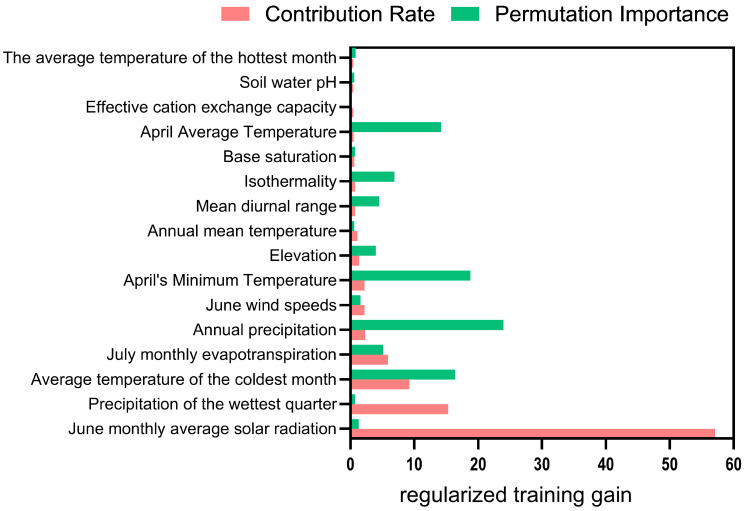
The environmental factors’ contribution rate and permutation importance index involved in modeling without human footprint.

Under natural conditions without human disturbance, among the 16 environmental factors used for modeling (Figure 8 and Supplementary Figure S1), the total contribution rates of the following factors reached 94.1%: average solar radiation in June (57.1%), precipitation of the wettest season (15.3%), mean temperature of the coldest month (9.2%), evapotranspiration in July (5.9%), annual precipitation (2.3%), average wind speed in June (2.2%), and minimum temperature in April (2.1%). These factors collectively emerged as the dominant environmental drivers influencing the distribution of suitable habitats for *Nitraria tangutorum*. The total importance rate of annual precipitation (24.0%), minimum temperature in April (18.8%), mean temperature of the coldest month (16.4%), average temperature in April (14.2%), isothermality (6.8%), evapotranspiration in July (5.1%), average diurnal range (4.5%), and altitude (4.0%) reach 93.8%, making them critical factors influencing the potential suitable habitat distribution of *Nitraria tangutorum*.

Based on the response curve of environmental factors (Supplementary Figure S2), the threshold values of environmental factors for the suitable habitats of *Nitraria tangutorum* are defined as follows:

- June average solar radiation: >23,696 kJ/(m^2^·day) , with an optimal value at 25,067 kJ/(m^2^·day) ;
- Wettest month precipitation: 3.7–83.7 mm, with an optimal value at 3.7 mm;
- Mean temperature of the coldest month: −34.7–−7.7 °C, with an optimal value at −29.5 °C;
- Annual precipitation: 6.2–144.7 mm, with an optimal value at 6.2 mm;
- April minimum temperature: 1.5–27.7 °C, with an optimal value at 23.0 °C;
- April mean temperature: 9.2–31.1 °C, with an optimal value at 26.4 °C.

### 3.4. Changes in Suitable Habitats of Nitraria tangutorum Under CMIP6 Climate Scenarios

**Figure 9:**
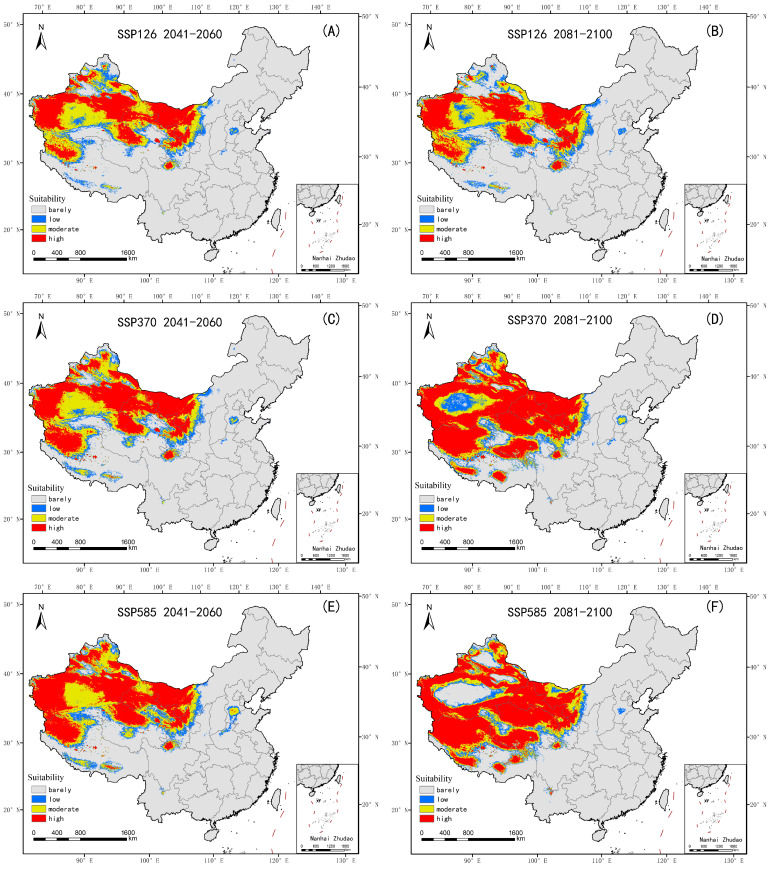
Classification of suitable areas for *Nitraria tangutorum* under different climatic scenarios. (**A**) 2041–2060 SSP126; (**B**) 2081–2100 SSP126; (**C**) 2041–2060 SSP370; (**D**) 2081–2100 SSP370; (**E**) 2041–2060 SSP585; (**F**) 2081–2100 SSP585.

Under the CMIP6 scenarios, the potential suitable habitat of *Nitraria tangutorum* shows a significant bidirectional development trend, migrating towards higher latitudes and elevations under different emission pathways (Figure 9), a trend that intensifies with the extension of the future climate scenarios. Compared to the current climate baseline period (1970–2000), the suitable habitat of *Nitraria tangutorum* expands significantly under all SSP emission pathways, with this expansion trend further intensifying in medium–high emission scenarios (SSP3-7.0) and extreme emission scenarios (SSP5-8.5) as the timeline extends. According to Supplementary Figure S3, (1) stable areas are concentrated in the arid and semi-arid regions of Northwestern China and parts of the Qinghai–Tibet Plateau; (2) extinct areas are primarily located near 110° E, and under the SSP5-8.5 emission scenario during the 2081–2100 period, significant contiguous extinction of suitable areas also occurs across major basins in Xinjiang; and (3) expanding areas are concentrated in higher-latitude and higher-altitude regions, and this trend is significantly intensified with increasing emission values and prolongation of the timeline.

**Figure 10:**
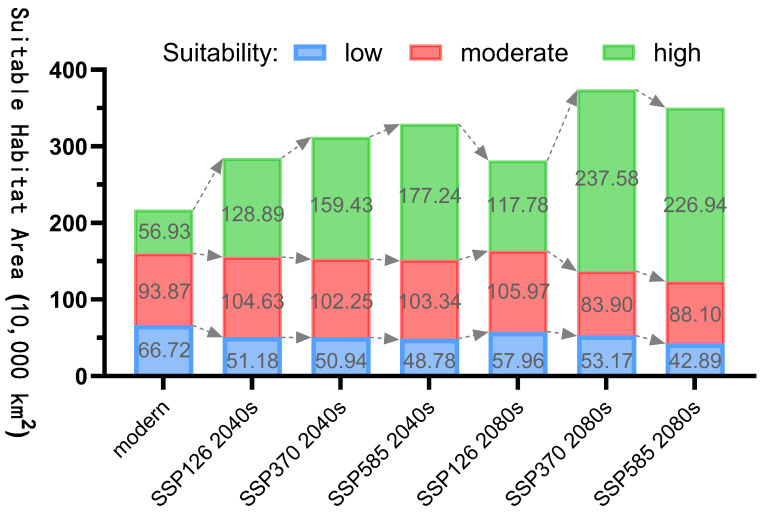
The habitat area changes for *Nitraria tangutorum* under future climate scenarios.

**Figure 11:**
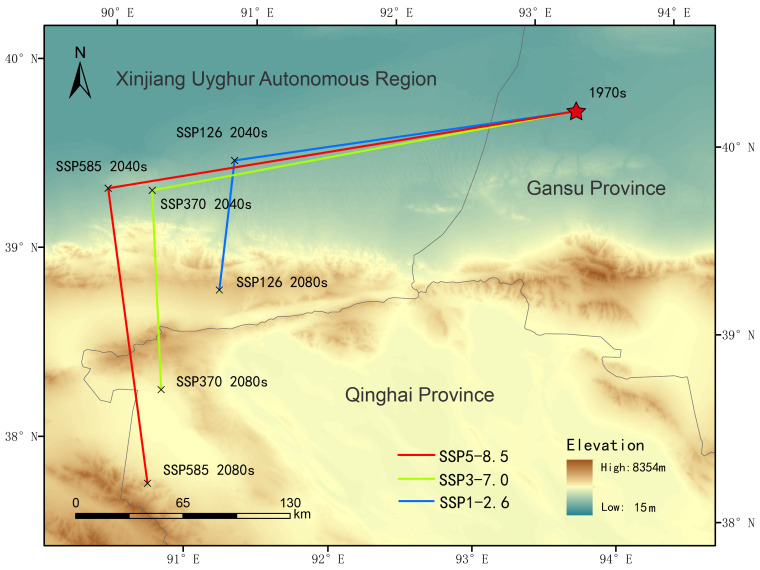
The changes in the distribution center of *Nitraria tangutorum* under future climate scenarios.

Figure 10 illustrates the trend of habitat area change for *Nitraria tangutorum* under different climate scenarios, while Figure 11 shows the variation in distribution center (centroid) of *Nitraria tangutorum* under future climate scenarios. The data demonstrate a significant expansion trend in the Highly Suitable area. Under the three emission scenarios (SSP1-2.6, SSP3-7.0, SSP5-8.5), from 2041 to 2060, the Highly Suitable area is projected to reach 1,288,900 km^2^ (126.41% increase), 1,594,300 km^2^ (180.06%), and 1,772,400 km^2^ (211.36%), respectively, showing a clear positive correlation between the spatial extent of the Highly Suitable area and the level of emissions. Subsequently, the centroids in the three emission scenarios exhibit a substantial westward shift, with the movement distance positively correlated with emission levels. In the high emission scenarios SSP3-7.0 and SSP5-8.5, from 2081 to 2100, the Highly Suitable area is forecasted to expand to 2,375,800 km^2^ (217.35% increase) and 2,269,400 km^2^ (198.66% increase), forming a nearly threefold scale of growth poles. In contrast, under the sustainable development model (SSP1-2.6), the Highly Suitable area in the distant-future-climate-scenario period (2081–2100) shows significant contraction compared to the near-future-climate-scenario period (2041–2060). In relation to the distant-future-climate-scenario period (2081–2100), the centroids in the three emission scenarios all shift southward (higher altitude), and the movement distance increases with the level of emission.

### 3.5. Multivariate Environmental Similarity Surfaces and Least Similar Variables Analysis

As shown in Supplementary Figure S4, under six emission scenarios—2041–2060 SSP1-2.6, 2041–2060 SSP3-7.0, 2041–2060 SSP5-8.5, 2081–2100 SSP1-2.6, 2081–2100 SSP3-7.0, and 2081–2100 SSP5-8.5—the mean Multivariate Environmental Similarity Surfaces (MESS) values across 260 modern distribution points of *Nitraria tangutorum* were 26.43, 23.99, 21.52, 26.17, 11.60, and 8.34, respectively, with negative similarity points accounting for 1.54%, 1.54%, 1.54%, 1.54%, 7.31%, and 20.00%. The results indicate that the 2081–2100 SSP5-8.5 scenario exhibits the highest climate anomaly levels (52 out of 260 points showed negative similarity), followed by 2081–2100 SSP3-7.0, while the 2041–2060 SSP1-2.6 scenario had the lowest anomalies. Spatially, climate anomalies under all 2041–2060 scenarios were confined to the Tuha Alluvial-Fan Alluvial Plain, driven by Bio10 (mean temperature of warmest month) as the most dissimilar variable (Supplementary Figure S5). In the 2081–2100 scenarios, the anomalies expanded as follows: SSP3-7.0 affected the Alxa Plateau and Xinjiang Basin, while SSP5-8.5 formed a trans-ecological continuous belt across the Alxa Plateau, Xinjiang, and Huang-Huai-Hai Alluvial Plain (near Hengshui), with Bio10 remaining the sole critical variable. These findings highlight Bio10’s dominant role in climate-driven habitat suitability projections.

## 4. Discussion

### 4.1. Changes in the Suitable Habitat of Nitraria tangutorum Under the Current Climate Baseline Period

This study constructed a niche model based on the maximum entropy principle, integrated with current environmental variables to conduct spatial predictions for the potential suitable habitat of *Nitraria tangutorum*. The model results showed an AUC value of 0.914 ± 0.0115, indicating strong predictive accuracy of the model predictions. The core suitable habitats of *Nitraria tangutorum* are distributed in regions such as the northern foothills of Yinshan in Inner Mongolia, the western edge of the Tarim Basin, the Hexi Corridor, and the Qaidam Basin. These spatial distribution patterns overlap with the predicted results of other scholars [6,36], and match 100% with multi-phase field investigation data from this laboratory. Furthermore, this study identified through comparative analysis that previous prediction studies on the geographical distribution of *Nitraria* have methodological limitations: regional models in China [52] are often constrained by modeling frameworks driven solely by individual natural environmental factors without considering social factors, while global-scale studies [35] are limited by an insufficient spatial distribution of sample points and significant differences in niche model assumptions. This mismatch between data–model coupling frameworks ultimately results in insufficient explanatory power for the spatial heterogeneity of cross-scale prediction results [43,53].

To address this, we incorporated the Human Footprint Index, representing human disturbances to further improve the accuracy of model predictions [54,55]. Human disturbances pose multi-scale threats to the survival of desert plants. Their impacts manifesting through processes including habitat compression, resource deprivation, and ecological corridor fragmentation, which directly lead to declines in population density, impeded gene flow, and degradation of habitat quality [56,57,58]. Considering the magnitude of human disturbances, the model’s AUC value increased from 0.888 to 0.914 (+0.026), significantly enhancing predictive precision, bringing the results closer to the actual species distribution. After adding the human footprint factor, the suitable habitat area for *Nitraria tangutorum* exhibited a substantial decrease: the overall suitable area decreased by 12.12% compared to the scenario without human disturbances, while the high-suitability and medium-suitability areas reduced by 40.02% and 41.17%, respectively. Under human disturbance, high-suitability habitats were geographically confined to the western alpine and extreme alpine areas of the Kunlun Range within the alpine basin–mountain zone of the Tibetan Plateau, as well as to the Hetao Alluvial Plain and the subalpine areas of Helan Mountain within the mid-eastern region of the North China–Inner Mongolia Highlands. The formation of this geographic distribution pattern is influenced by two driving factors: (1) ecological vulnerability in desert core areas, where sparse native vegetation created natural niche availability, while human activities expanded seed dispersal distances; and (2) spatial extrapolation bias in original models overestimated suitability in arid regions [59], which was corrected by incorporating human activity parameters. Post-correction predictions showed high congruence with typical desert zones (annual precipitation <100 mm), where extreme aridity aligns with *Nitraria tangutorum*’s water-use strategy, making it a priority species for desertification control.

### 4.2. Impacts of Future Climate Scenario Change on the Suitable Habitat of Nitraria tangutorum

Under the background of future climate scenario changes, the spatial pattern of the potential suitable habitat of *Nitraria tangutorum* is undergoing significant reorganization. Future climate scenario simulation based on the MaxEnt model indicates that increases in annual temperature range and mean temperature of the warmest month (Bio10) thresholds (two-factor percentile intervals reaching 23.5 and 16.0, respectively) have become the key climatic factors determining the boundary shift of its distribution. This has resulted in the migration of suitable habitats towards higher altitudes (average annual elevation increase: 12.7 m/year) and higher latitudes (average annual eastward shift: 0.8°). This migration trend of *Nitraria tangutorum* is also reflected in another desert plant, *Alhagi sparsifolia* Shap. and *Hippophae rhamnoides* subsp. *yunnanensis* L. [53,60].

Compared to the current climate baseline period (1970–2000), the potential suitable habitat for *Nitraria tangutorum* exhibits significant expansion under all SSP emission pathways, with this expansion trend further intensifying over time under high-emission scenarios (SSP3-7.0) and extreme emission scenarios (SSP5-8.5). Contrary to the shrinking trends of suitable habitats for typical desert plants such as *Haloxylon ammodendron* and *Tamarix ramosissima* under future climate scenarios [61,62], *Nitraria tangutorum* demonstrates a significant potential for expansion under SSP3-7.0 to SSP5-8.5 scenarios. Predictions by other scholars on the overall future climate scenario distribution of *Nitraria* also confirm the expansion trend of *Nitraria* under future climate scenarios [6].

It is worth noting that under SSP3-7.0 and SSP5-8.5 scenarios, significant gaps in *Nitraria tangutorum* potential suitable habitats were identified in the northwestern basins during 2080–2100. This indicates that extreme climate conditions (mean temperature of the warmest month anomalies) in these scenarios exceed ecological thresholds, making the region unsuitable for species persistence. Our analysis using Multivariate Environmental Similarity Surfaces (MESS) and least similar variable identification confirmed that the critical divergence in July’s mean temperature (Bio10) between the climate scenarios and the current climate baseline period is the primary driver of these habitat gaps. Supporting this finding, a prior study on climate projections in Xinjiang under SSP5-8.5 revealed spatially heterogeneous warming rates, with the Junggar Basin and Tarim Basin experiencing temperature increases of 0.72–0.76 °C per decade, 116–137% higher than regional averages [63].

However, this study also has limitations, such as neglecting species dispersal mechanisms and failing to quantify population propagule migration. We hope that future studies can further deepen research in this direction from the perspective of population genetics.

### 4.3. Spatial Response Patterns of Suitable Habitats to Climate Scenario Variations

**Figure 12:**
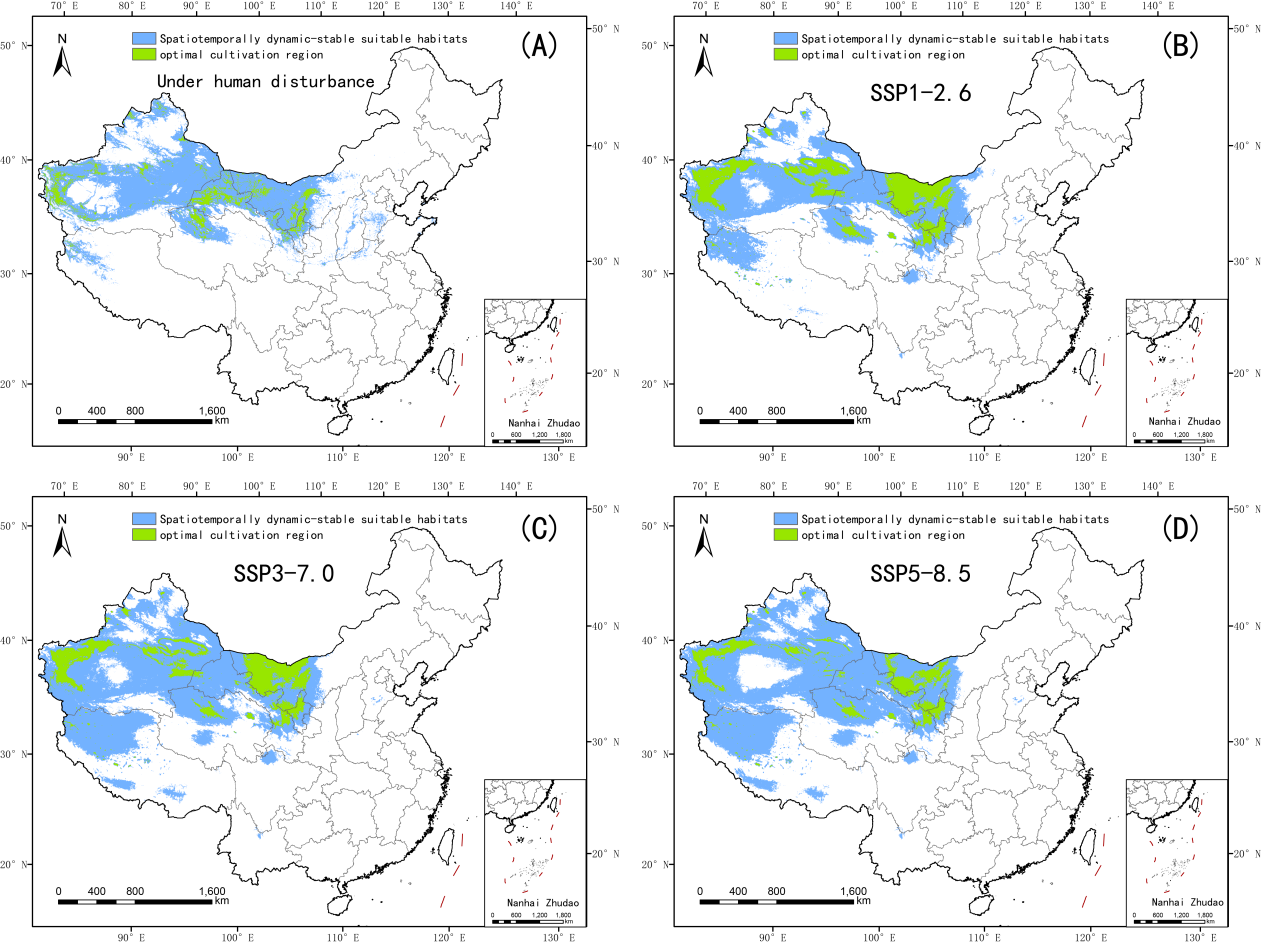
Stable suitable habitat ranges for *Nitraria tangutorum* under different scenarios. (**A**–**D**) The stable distribution ranges of ecologically suitable habitats under four scenarios: human disturbance, SSP1-2.6, SSP3-7.0, and SSP5-8.5.

Figure 12 illustrates the dynamic distribution characteristics of optimal cultivation regions of *Nitraria tangutorum* under the influence of human activities or climate change through four subfigures (A–D). Subfigure A focuses on the human disturbance scenario, revealing that the stable suitable habitats are concentrated in five provincial-level regions: Inner Mongolia Autonomous Region, Xinjiang Uygur Autonomous Region, Gansu Province, Qinghai Province, and Ningxia Autonomous Region, with sporadic patch distributions also present in northwestern Tibet. The optimal cultivation regions remain only in areas such as the western part of the Hetao Plain, the Helan Mountain region, and high mountainous areas of western Kunlun, far from urban regions in the alluvial and diluvial plains of the Hexi Corridor, among others. Spatial analysis indicates that human disturbances have caused a 40.02% reduction in the area of optimal cultivation regions compared to the current climate baseline period (1980–2020), driven by urbanization-based cropland conversion, overgrazing, and land-use fragmentation. Subfigures B–D depict climate scenario impacts: under SSP1-2.6 (low–medium emission), SSP3-7.0 (medium–high emission), and SSP5-8.5 (extreme emission)—stable habitats expand northwestward while optimal zones contract sharply. Under SSP5-8.5, optimal zones diminish by 40.6% relative to medium–low emission scenarios (SSP3-7.0) and 34.5% compared to SSP1-2.6, retreating to high-altitude refugia (≥3500 m) with minimal overlap with stable zones. This spatial decoupling underscores climate change-induced regime shifts, where extreme emissions exacerbate habitat vulnerability despite nominal expansion in low-emission future climate scenarios.

### 4.4. Influencing Factors: Environmental Variables Determining Species Distribution Patterns

In this study on predicting the suitable habitat of *Nitraria tangutorum* in the current climate baseline period, solar radiation was identified as a critical environmental factor influencing its distribution, with its importance in the modeling of natural environmental factors exceeding the combined importance of all other factors. Other research on the distribution of *Nitraria* has similarly demonstrated the strong importance of solar radiation (UV), accounting for 65.5% of explanatory power [35]. Studies indicate that solar radiation intensity during the species’ growing season exhibits significant seasonal variation, with radiation intensity peaking in June. This temporal pattern drives critical thermodynamic thresholds for photosynthetic carbon assimilation and elevates photon flux density, enhancing photosystem II efficiency. Approximately 50% of daytime net radiation is converted into sensible heat via atmospheric turbulence, which elevates ambient air temperature. This energy transfer mechanism not only optimizes leaf energy conversion but also amplifies stomatal conductance-mediated transpiration, thereby strengthening root uptake of deep soil moisture through cross-scale hydrological coordination. Such energy–water coupling provides the eco-hydrological driving force for maintaining stable “root–canopy” water cycling [64,65].

Research results further indicate that precipitation factors have a significant impact on the ecological adaptability model, with the precipitation of the wettest season contributing 15.3% and the annual precipitation index contributing 24.0%. However, the ecological adaptability threshold of this species to moisture conditions is extremely narrow, specifically reflected in the adaptation range of precipitation in the wettest season being 3.7–83.7 mm, the annual precipitation adaptation range being 6.2–144.7 mm, and the optimal moisture conditions being at the lower limit of the thresholds (3.7 mm and 6.2 mm). This narrowing niche specificity reduces interspecies competition under arid conditions. Evidence confirms that in desert ecosystems with annual precipitation <150 mm, *Nitraria tangutorum* can form mono-dominant communities through its unique physiological and ecological adaptations (such as deep root systems for groundwater absorption and stomatal regulation mechanisms to reduce water loss), thereby becoming the most widely distributed plant functional group in such extreme habitats [66,67]. Research on the eco-hydrological driving mechanism of the root–crown water cycle in *Nitraria tangutorum* has provided new insights into its arid ecological characteristics [68].

The geographical distribution pattern of *Nitraria tangutorum* under natural conditions exhibits a significant dependence on low-temperature environments. Sensitivity analysis of the model indicates that the substitution contribution of low-temperature factors (mean temperature of the coldest month and extreme low temperatures in spring) reaches 35.2%, notably higher than precipitation factors (24.7%) and soil factors (1.2%). Previous ecological niche modeling studies on *Nitraria roborowskii* and *Nitraria sphaerocarpa* have also confirmed that tolerance to low-temperature stress is a key limiting factor for their distribution [69]. This situation is not unique, as dependency on low-temperature environments is also distinctly evident in other desert species like *Alhagi sparsifolia* [35].

## 5. Conclusions

This study constructed an HF-MaxEnt coupled model by integrating the Human Footprint Index with the MaxEnt niche model, innovatively overcoming the limitations of traditional niche models in characterizing the impacts of human activities. Through multi-factor coupling, the model’s AUC value was increased by 0.03, and its fitting accuracy reached an “Excellent predictive performance” level. Further insights revealed that June solar radiation, maximum precipitation in the rainy season, and mean temperature in the coldest month constitute the core driving factors for the distribution of *Nitraria tangutorum*. Additionally, the study found that human activities have led to a degradation rate of 79.4% in the highly suitable areas of *Nitraria tangutorum*, underscoring urgent ecological pressures requiring attention. Future climate scenario simulations indicated that under high-intensity carbon emission scenarios, the total area of suitable habitats for *Nitraria tangutorum* is projected to expand over 150%. Spatial migration will exhibit dual characteristics: “southwest gradient progression” and “high-altitude vertical migration”. However, the mean temperature in the hottest month will emerge as the core climatic limiting factor under this scenario, with significant habitat gaps anticipated in ecologically fragile regions such as the Tarim Basin in southern Xinjiang.

These findings not only enhance the theoretical framework for understanding the climatic adaptability of desert plants through the integration of multi-source environmental data and cutting-edge ecological models but also provide actionable decision-making support for constructing ecological security barriers in arid regions. Future research could focus on deepening the quantitative association mechanisms between social factors and species distribution, analyzing the direct or indirect impact pathways of human activities on desert plants like *Nitraria tangutorum*, or conducting climate key factor threshold tests based on migration characteristic predictions to further validate the ecological feasibility of these migration trends.

## Figures and Tables

**Table 1:** Screening environmental factor data.

Variable Type	Variable Name	Variable Description	Unit	Original Spatial Resolution
Temperature	bio1	Annual mean temperature	°C	30 arc-s
	bio2	Monthly mean diurnal temperature range	°C	
	bio3	Isothermality		
	bio10	Mean temperature of warmest month	°C	
	bio11	Mean temperature of coldest month	°C	
	tavg4	April mean temperature	°C	
	tmin4	April minimum temperature	°C	
Precipitation	bio12	Annual precipitation	mm	30 arc-s
	bio16	Wettest month precipitation	mm	
Evaporation	evam7	July evapotranspiration	mm/month	30 arc-s
Radiation	srad6	June mean solar radiation	kJ/(m^2^·day)	30 arc-s
Wind	wind6	June mean wind speed	m/s	30 arc-s
Soil	bsat	Base saturation	%	30 arc-s
	ph_water	Water pH	−log H^+^	
	cec_eff	Effective cation exchange capacity	cmol/kg	
Topography	eleva	Elevation	m	30 arc-s
Human Impact	HF	Human footprint		30 arc-s

**Table 2:** Current and future environmental factor data selection.

Variable Name	Variable Description	Unit
bio1	Annual mean temperature	°C
bio2	Monthly mean diurnal temperature range	°C
bio3	Isothermality	
bio4	Temperature variation coefficient	
bio7	Temperature annual range	°C
bio10	Mean temperature of warmest month	°C
bio11	Mean temperature of coldest month	°C
bio12	Annual precipitation	mm
bio15	Precipitation seasonality	
bio16	Wettest month precipitation	mm

**Table 3:** Changes in the suitable growth areas of *Nitraria tangutorum* across the national range.

Category	Area (×10^4^ km^2^)		Change Under HD (×10^4^ km^2^)	Change Rate
	Without HD	Under HD		
Suitable Area	224.82	197.58	−27.24	−12.12%
Lowly Suitable Area	75.44	129.38	53.94	71.49%
Moderately Suitable Area	99.01	57.84	−41.17	−41.58%
Highly Suitable Area	50.37	10.35	−40.02	−79.44%

Without HD and Under HD represent without human disturbance and under human disturbance in sequence.

## Data Availability

Data are contained within the article.

## Supplementary Materials

The following supporting information can be downloaded at https://www.mdpi.com/article/10.3390/plants14172706/s1, Figure S1: Jackknife test of the environmental factors involved in modeling without human footprint; Figure S2: Response curves of dominant environmental drivers without human interference; Figure S3: Analysis of spatial superposition of *Nitraria tangutorum* Bobr. suitable areas under different climate scenarios; Figure S4: Multivariate Environmental Similarity Surfaces (MESS) of *Nitraria tangutorum* Bobr. predicted by the Maximum Entropy Model under different emission scenarios; Figure S5: The most dissimilar variable (Mod) of *Nitraria tangutorum* Bobr. predicted by the Maximum Entropy Model under different emission scenarios.

## Abbreviations

The following abbreviations are used in this manuscript:

RM	Regularization Multiplier
FC	Feature Combination
ROC	Receiver Operating Characteristic Curve
AUC	Area Under The Receiver Operating Characteristic Curve
MTSPS	Maximum Test Sensitivity and Specificity
HSIs	Habitat Suitability Indices
MESS	Multivariate Environmental Similarity Surfaces
MOD	Most Dissimilar Variables Analysis
HD	Human Disturbance
HF	Human Footprint Index

## References

[1] Zhao Y.L., Zhang L.J. (1998). Research on Quantitative Evaluation Methods for Vulnerable Ecological Environments. Adv. Geogr. Sci..

[2] Wang G.X., Cheng G.D., Xu Z.M. (1999). Water resource utilization and its ecological and environmental issues in the arid region of northwest China. J. Nat. Resour..

[3] Tuerxun M. (2017). Analysis and Research on the Suitable Habitat of *Xanthium italicum* in Xinjiang. Master’s Thesis.

[4] Wang Y., Sun Y., Hu T., Qin D.H., Song L.C. (2018). Attribution of Temperature Changes in Western China. Int. J. Climatol..

[5] Wang Y.G., Yang X.H., Yu C.T., Hu Z.S. (2007). Current status, ecological functions, and conservation strategies of Nitraria plants. J. Soil Water Conserv. Res..

[6] Duan Y.Z., Zhu G.X., Du Z.Y., Li Y., Lu K., Shi J.G. (2021). Simulated analysis of suitable habitats for *Nitraria* Plants Arid Reg. Northwestern China. Arid Zone Resour. Environ..

[7] Yu S.F., Mai M.T., Aysilemiti A.S. (2016). A review on research of Nitraria plants in Xinjiang. J. Agric. Sci..

[8] Zhu L.M., Wu J.X., Li M.J., Fang H., Zhang J.B., Chen Y.C., Chen J.H., Cheng T.L. (2023). Genome-Wide Discovery of CBL Genes in *Nitraria tangutorum* Bobr. and Functional Analysis of *NtCBL1-1* under Drought and Salt Stress. For. Res..

[9] Li H., Zhang Y.C., Zhang P. (2006). Review on *Nitraria* L. Species Research. J. Agric. Sci. Res..

[10] Chinese Academy of Sciences, Editorial Committee of the Flora of China (2004). Flora of China.

[11] Li S.F., Zhang Q.C., Zhang Q.C., Zong C.W., Tian X.F. (2005). Research progress on Nitraria plants. J. Beihua Univ. (Natural Sci. Ed.).

[12] Jiang X. (2003). Correlation Between Geographical Distribution of Multiple Plant Species and Climate in Arid Northwestern China and Prediction of Potential Distributions. Master’s Thesis.

[13] Li X.Z. (2014). Impact of Climate Change on Growth and Development of Dominant Forage Grasses in Inner Mongolia Grasslands. Master’s Thesis.

[14] Ren L.C. (2011). Introduction and Drought-Cold Tolerance Study of 13 Woody Plant Species in Baiyunebo Mining Area. Master’s Thesis.

[15] Du J.H., Yan P., Dong Y.X. (2010). Phenological Response of *Nitraria tangutorum* Bobr. to Climate Change in Minqin County, Gansu Province, Northwest China. Int. J. Biometeorol..

[16] Yu S.F., Ai S.D., Li M.T. (2015). Correlation analysis of biomass in *Nitraria tangutorum* Bobr. Shrubs Its Predict. Model. West. For. Sci..

[17] Jiang F.Z., Wang J., Zhang Y.P. (2005). Survey and comprehensive utilization of wild Nitraria resources in the Qaidam Basin. Qinghai Sci. Technol..

[18] Ji D.J., Zhang D.F., Yu Q. (2022). Investigation on germplasm resources of Nitraria in the Qaidam Basin and their utilization prospects. Qinghai Agric. For. Sci. Technol..

[19] Bai M.S., Li G.Q., Chen Y.Y. (2008). Regional study on medicinal active ingredient content in Nitraria. J. Northwest For. Univ..

[20] Zhang P., Ha S., Yue X.L., Zhuang Y.M. (2008). Morphology and sedimentary characteristics of *Nitraria* Shrub Sand Dunes. J. Arid Land Geogr..

[21] Zhang H.C., Li S.H., Mason J.A., Yizhaq H., Gui D.W., Xu Z.W. (2024). Biogeomorphological niche of a landform: Machine learning approaches reveal controls on the geographical distribution of *Nitraria tangutorum* Bobr. Nebkhas. Earth Surf. Process. Landforms.

[22] He Y.H., Tian Y.L., Ye D.M., Qin J.Q., Guo L.S. (2005). Relationships between aboveground biomass and leaf area in Nitraria plants. Chin. Desert.

[23] Qiu G.Y., Li C., Yan C. (2015). Characteristics of soil evaporation, plant transpiration, and water budget in Nitraria dunes in arid northwest China. Gricultural For. Meteorol..

[24] Li X.L., Dang X.H., Zhai B., Wei Y.J., Chi X., Wu H.M. (2022). Architectural Characteristics and Biomass Allocation Patterns of Adventitious Roots in *Nitraria tangutorum* Shrubland. Chin. J. Deserts.

[25] Xu S.Q., Ji X.B., Jin B.W., Zhang J.B. (2016). Root distribution of three dominant desert shrubs and their water uptake dynamics. J. Plant Ecol..

[26] Zhu L.H., Fang Z.K., Suo Y.R. (2005). Characteristics and development prospects of *Nitraria tangutorum* Qaidam Basin. Qinghai Sci. Technol..

[27] Gao H., Suo Y.R. (2002). Amino acid contents and nutritional evaluation of *Nitraria sibirica* and *Nitraria tangutorum* Qaidam Basin. Amino Acids Biol. Resour..

[28] Ma H. (2014). Study on Bioactive Components in *Nitraria tangutorum* Bobr. Doctoral Dissertation.

[29] Soberón J.M. (2010). Niche and area of distribution modeling: A population ecology perspective. Ecography.

[30] Zhu G.P., Liu G.Q., Bu W.J., Gao Y.B. (2013). Fundamental principles of ecological niche models and their applications in biodiversity conservation. Biodiversity.

[31] Elith J., Graham C.H., Anderson R.P., Dudίk M., Ferrier S., Guisan A., Hijmans R.J., Huettmann F., Leathwick J.R., Lehmann A. (2006). Novel methods improve prediction of species’ distributions from occurrence data. Ecography.

[32] Chilimbi T., Hirzel M. (2002). Dynamic hot data stream prefetching for general-purpose pro-grams. ACM Sigplan Not..

[33] Hirzel A.H., Hausser J., Chessel D., Perrin N. (2002). Ecological-niche factor analysis: How to compute habitat-suitability maps without absence data?. ECOLOGY.

[34] Hirzel A., Guisan A. (2002). Which is the optimal sampling strategy for habitat suitability modelling. Ecol. Model..

[35] Lu K., Liu M., Feng Q., Liu W., Zhu M., Duan Y.Z. (2025). Pdicting the Global Distribution of *Nitraria* L. Under Climate Change Based on Optimized MaxEnt Modeling. Plants.

[36] Halimujiang M. (2021). Geographical Distribution Patterns and Study on Potential Suitable Habitats of four Nitraria Species. Master’s Thesis.

[37] Stanton J.C., Pearson R.G., Horning N., Ersts P., Akcakaya H.R. (2012). Combining static and dynamic variables in species distribution models under climate change. Methods Ecol. Evol..

[38] Dang R.L., Xiao L.P., Xue F.G. (2002). Phytogeographical Analysis of Plant Genera in the Arid Deserts of Northwestern China. Guangxi Plants.

[39] Warren D.L., Glor R.E., Turelli M. (2010). ENMTools: A toolbox for comparative studies of environmental niche models. ECOGRAPHY.

[40] Zhang Y.F., Chen S.T., Gao Y., Yang L., Yu H. (2023). Prediction of global potential suitable habitats of Nicotiana alata Link et Otto based on MaxEnt model. Sci. Rep..

[41] Mu H.W., Li X.C., Wen Y.N., Huang J.X., Du P.J., Su W., Miao S.X., Geng M.Q. (2022). A global record of annual terrestrial Human Footprint dataset from 2000 to 2018. Sci. Data.

[42] Zhu G.P., Qiao H.J. (2016). Impact of MaxEnt model complexity on predicting potential distribution ranges of species. Biodiversity.

[43] Yang J.T., Huang Y., Jiang X., Chen H., Liu M., Wang R.L. (2022). Potential geographical distribution of the edangred plant Isoetes under human activities using MaxEnt and GARP. Glob. Ecol. Conserv..

[44] Shen Y.F., Tu Z.H., Zhang Y.L., Zhong W.P., Xia H., Hao Z.Y., Zhang C.G., Li H.G. (2022). Predicting the impact of climate change on the distribution of two relict Liriodendron species by coupling the MaxEnt model and actual physiological indicators in relation to stress tolerance. J. Environ. Manag..

[45] Cobos M.E., Peterson A.T., Barve N., Osorio-Olvera L. (2019). kuenm: An R package for detailed development of ecological niche models using Maxent. PeerJ.

[46] Phillips S.J., Anderson R.P., Dudίk M., Schapire R.E., Blair M.E. (2017). Opening the black box: An open-source release of Maxent. ECOGRAPHY.

[47] Urbani F., D’Alessandro P., Frasca R., Biondi M. (2015). Maximum entropy modeling of geographic distributions of the flea beetle species endemic in Italy (Coleoptera: Chrysomelidae: Galerucinae: Alticini). Zool. Anz..

[48] Phillips S.J., Anderson R.P., Schapire R.E. (2006). MMaximum entropy modeling of species geographic distributions. Ecol. Model..

[49] Nolan V., Kaky E.D., Alatawi A.S., Gilbert F. (2022). Mapping the Indian crested porcupine across Iraq: The benefits of species distribution modelling when species data are scarce. Mamm. Biol..

[50] Li Y., Zhang X., Fang Y. (2016). esponse of spatial distribution patterns of Quercus chenii to climate change since the Last Glacial Maximum. J. Plant Ecol..

[51] Zhang X.Q. (2018). Geographical Distribution and Climate Suitability of Typical Ecologically and Economically Important Tree Species in Arid Northwest China. Doctoral Dissertation.

[52] Jiang S.X., Hu J., Wang F.L. (2024). Analysis of population dynamics of *Nitraria tangutorum* China Using MaxEnt Model. Chin. Wild Plant Resour..

[53] Yin H., Tian C., Ma Q.Q., Lv G.H., Zeng F.J. (2022). Spatiotemporal dynamics of potential distribution patterns of *Alhagi sparsifolia* under climate change and human disturbance. Acta Ecol. Sin..

[54] Anderson R.P., Peterson A.T., Gómez-Laverde M. (2002). Using niche-based GIS modeling to test geographic predictions of competitive exclusion and competitive release in South American pocket mice. OIKOS.

[55] Pan S.Y., Zhu Z.H., Yao T.H., Wang Y.X., Zhou P. (2016). Spatial distribution prediction of *Polygonum multiflorum* under climate change in China. J. Northwest A&F Univ. (Natural Sci. Ed.).

[56] Planillo A., Malo G.E. (2018). Infrastructure features outperform environmental variables explaining rabbit abundance around motorways. Ecol. Evol..

[57] Ma T.X., Liang Y., Li Z.Y., Liu B., Wu M.M., Lau M.K., Feng Y. (2023). Projected effects of climate change and urban expansion on species-level biodiversity of plants in main city clusters of Northern China. Front. Ecol. Evol..

[58] Yang Y.Y., Liu L.Y., Shi P.J., Zhang G.M., Qu Z.Q., Tang Y., Lei J., Wen H.M., Xiong Y.Y., Wang J.P. (2015). Morphology, spatial pattern and sediment of *Nitraria tangutorum* Nebkhas Barchans Interdune Areas Southeast Margin Badain Jaran Desert, China. Geomorphology.

[59] Elith J., Phillips S.J., Hastie T., Dudík M., Chee Y.E., Yates C.J. (2011). A statistical explanation of MaxEnt for ecologists: Statistical explanation of MaxEnt. Divers. Distrib..

[60] He M.Y., Ma F.Y., Ding J.J., Niu P.X., Luo C.K., Wang M., Jiang P. (2025). Analysis of Spatial Suitable Habitats of Four Subspecies of *Hippophae rhamnoides* China Based MaxEnt Model. Plants.

[61] Zhu N., Guo Y.L. (2023). Study on the distribution of suitable habitats for *Haloxylon ammodendron* (C.A.Mey) Bunge based on four commonly used machine learning algorithms. Ecol. Sci..

[62] Wu J.G., Lv J.J., Zhou Q.F. (2010). Potential impacts of climate change on the distribution of six desert plant species. Acta Bot. Sin..

[63] Zhang Y.L., Zhang P., Gu X.C., Long A.H. (2024). Projections of temperature and precipitation changes in Xinjiang from 2021 to 2050 based on the CMIP6 model. PLoS ONE.

[64] Ji X.B., Zhao W.Z., Jin B., Liu J., Xu F.N., Zhou H. (2021). Seasonal variations in energy exchange and evapotranspiration of an oasis-desert ecotone in an arid region. Hydrol. Process..

[65] Xu S.Q., Yu Z.B., Ji X.B., Edward A.S. (2017). Comparing three models to estimate transpiration of desert shrubs. J. Hydrol..

[66] Chun L., Na R.S., Zhao S.Z., Hao Y.Z., Hu Z.M., Ma Y.H. (2016). Resources and utilization of *Nitraria* Species. Chin. J. Wild Plant Resour..

[67] Yang H. (2021). Plant Diversity and Influencing Factors in the Kumtag Desert. Doctoral Dissertation.

[68] Qin J., Si J.H., Jia B., Zhao C.Y., Zhou D.M., He X.H., Wang C.L., Zhu X.L. (2023). Water use strategies of *Nitraria tangutorum* in the lake-basin region of the Badain Jaran Desert. Front. Plant Sci..

[69] Li X.C. (2018). Geographical Distribution Patterns of Major Desert Plants in Temperate China. Doctoral Dissertation.

