# Supplementary material for: Spatiotemporal Dynamics of Potential Distribution Patterns of Nitraria tangutorum Bobr. Under Climate Change and Anthropogenic Disturbances
Source: Plants (Basel). 2025 Aug 30;14(17):2706. doi: 10.3390/plants14172706 (PMC12430191; doi:10.3390/plants14172706)
Supplement: Supplementary file 1 [file plants-14-02706-s001.zip › plants-3769416-supplementary.pdf]

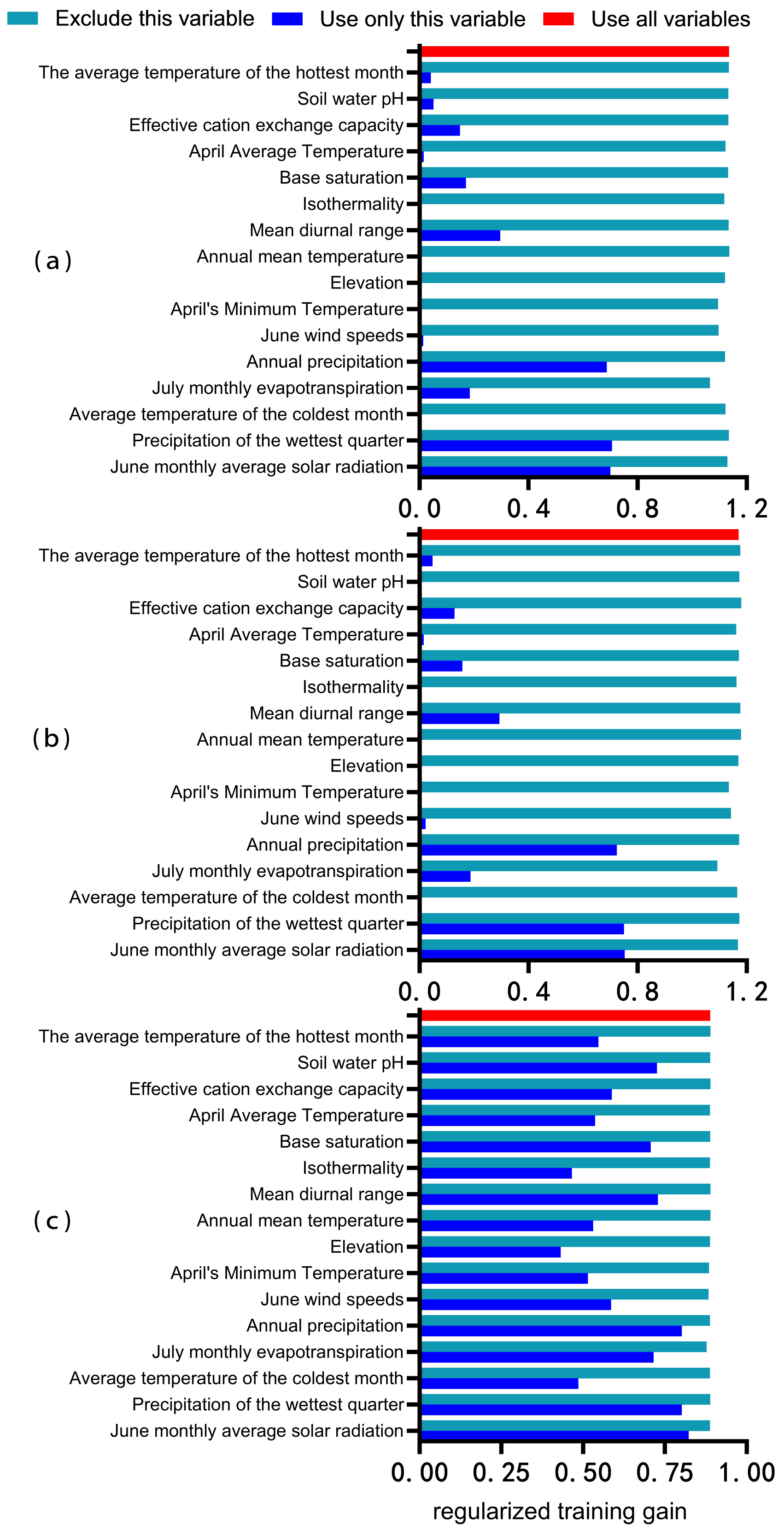

Sup Figure S1: Jackknife test of the environmental factors involved in modeling without human footprint. A, B, C represent regularized training gain, testing gain, and area under the receiver operating characteristic curve.

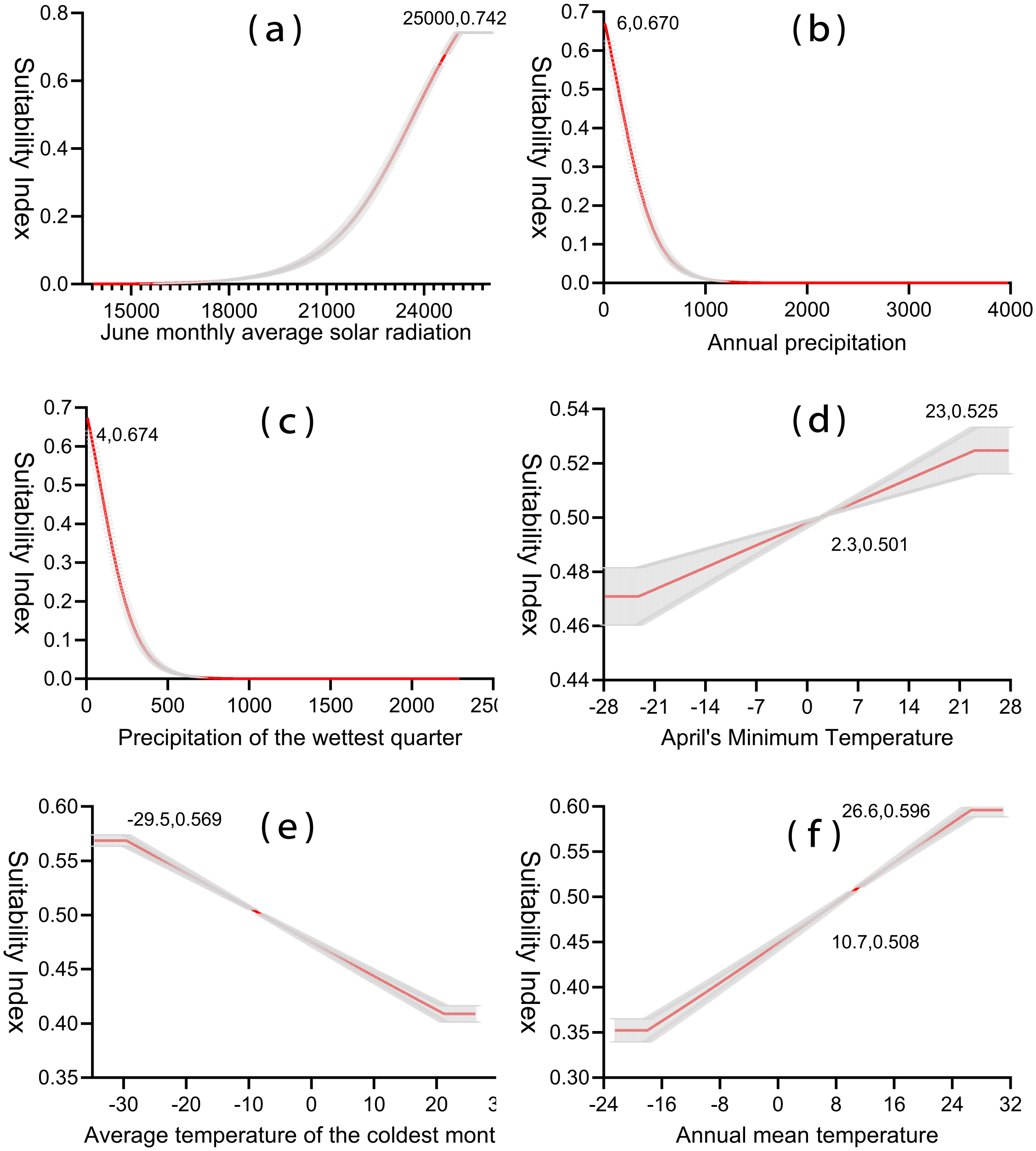

Sup **Figure** S2: Response curves of dominant environmental drivers without human interference. A, B, C, D, E, F represent the response curves for environmental factors including June Average Solar Radiation, Annual Precipitation, Wettest-Season Precipitation, April Minimum Temperature, Coldest-Month Average Temperature and April Mean Temperature.

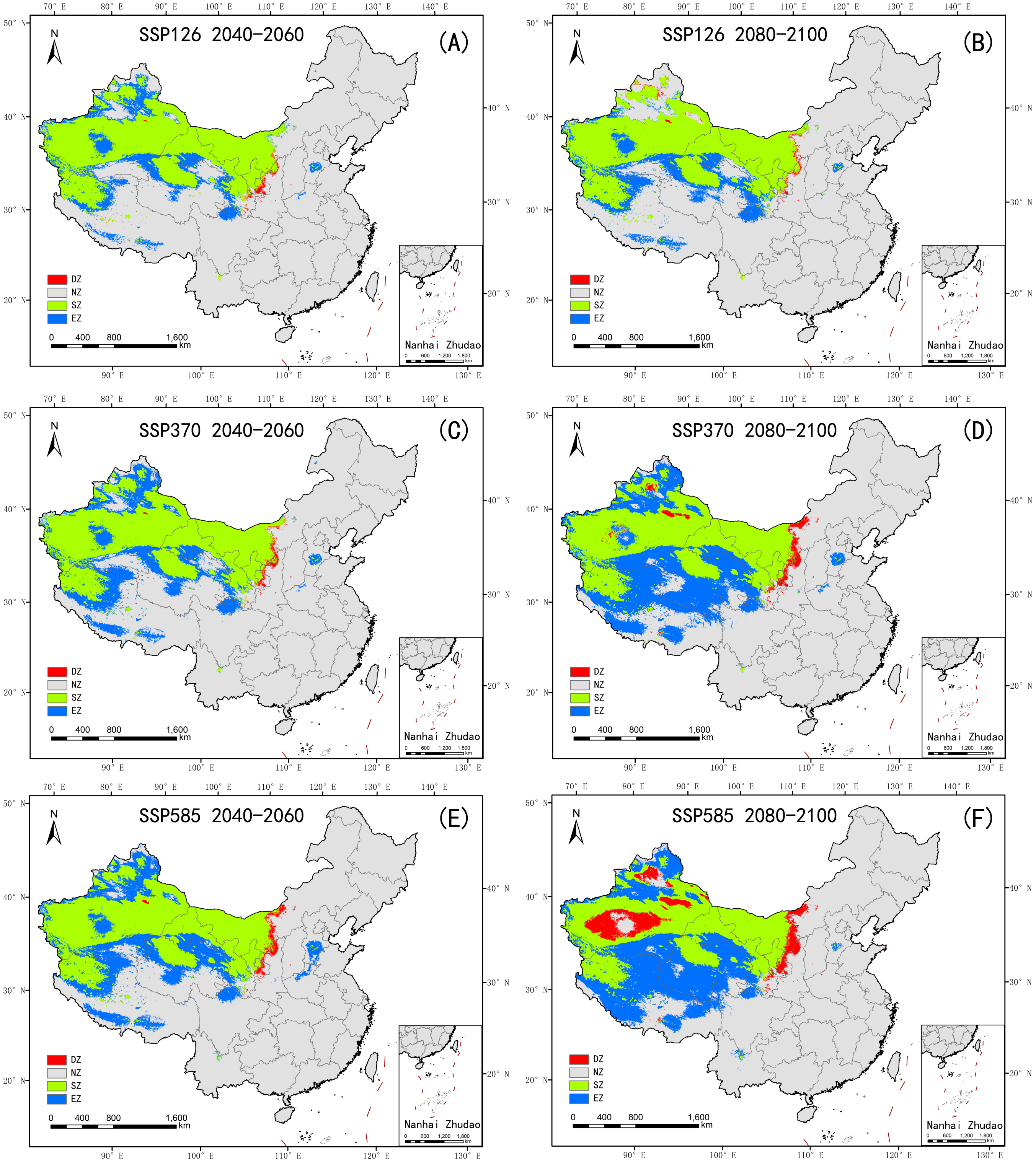

Sup Figure S3: Analysis of spatial superposition of *Nitraria tangutorum* Bobr. suitable areas under different climate scenarios. A, 2041-2060 SSP126; B, 2081-2100 SSP126; C, 2041-2060 SSP370; D, 2081-2100 SSP370;E, 2041-2060 SSP585; F, 2081-2100 SSP585.NZ, DZ, SZ and EZ represent Non-suitable Zone, Declining Habitat Zone, Static Ecological Zone and Habitat Expansion Zone in sequence.

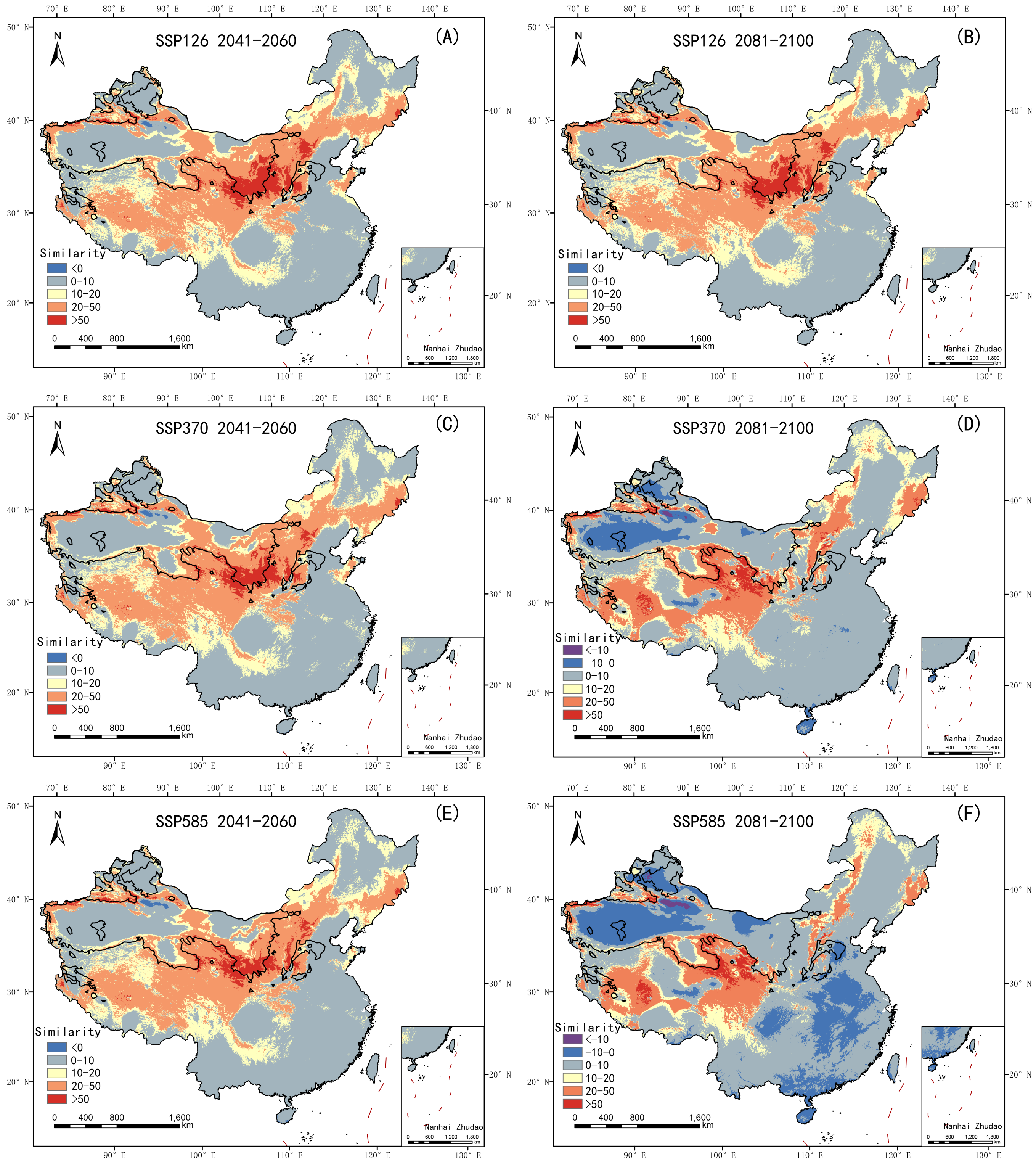

Sup **Figure** S4: Multivariate Environmental Similarity Surface (Mess) of *Nitraria tangutorum* Bobr. Predicted by the Maximum Entropy Model Under Different Emission Scenarios. A, 2041-2060 SSP126; B, 2081-2100 SSP126; C, 2041-2060 SSP370; D, 2081-2100 SSP370;E, 2041-2060 SSP585; F, 2081-2100 SSP585. The black-bordered regions represent the potential suitable habitat of *Nitraria tangutorum* during the current period (1970-2000).

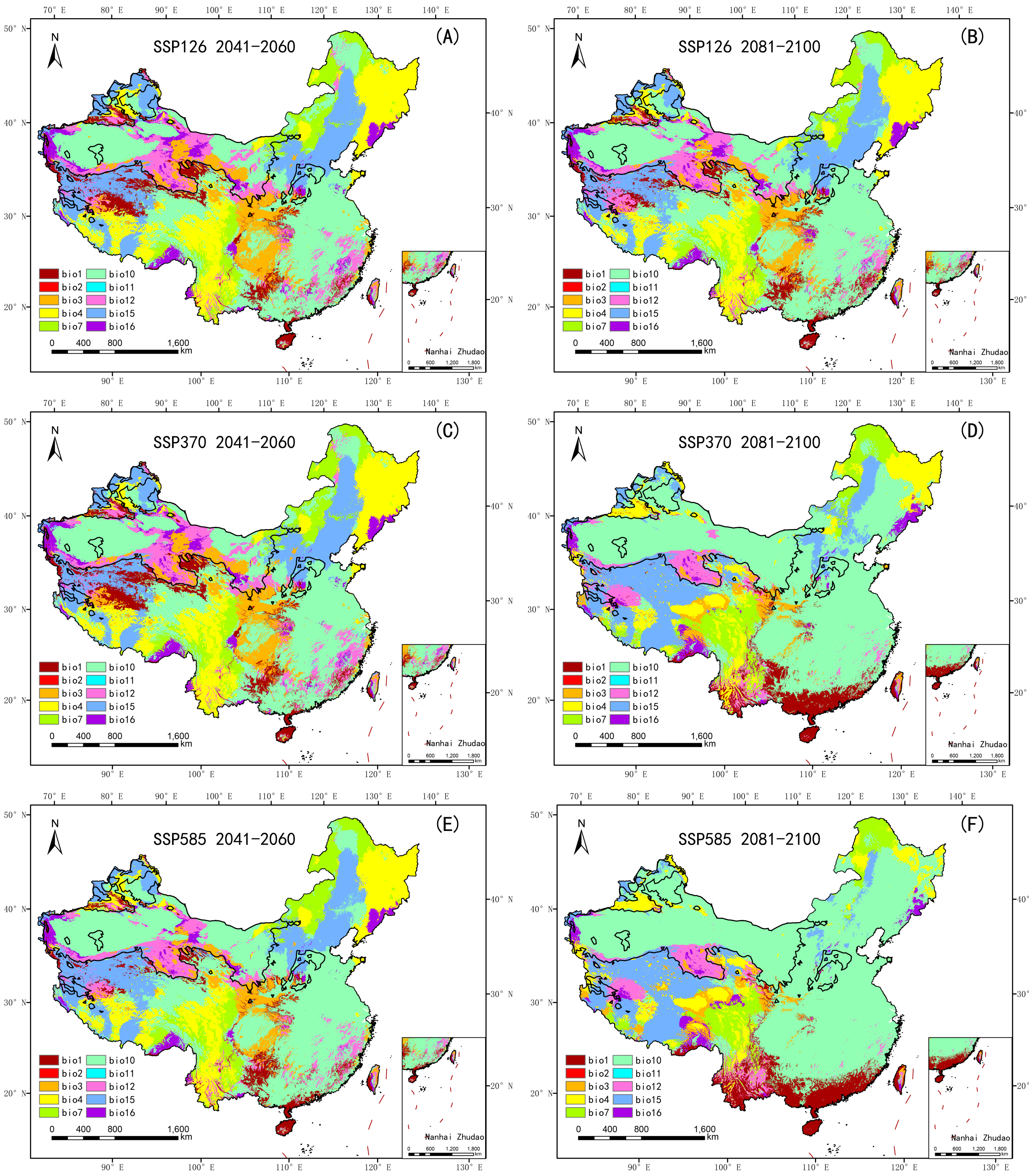

Sup **Figure** S5: The most dissimilar variable (Mod) of *Nitraria tangutorum* Bobr. Predicted by the Maximum Entropy Model Under Different Emission Scenarios. A, 2041-2060 SSP126; B, 2081-2100 SSP126; C, 2041-2060 SSP370; D, 2081-2100 SSP370;E, 2041-2060 SSP585; F, 2081-2100 SSP585. The black-bordered regions represent the potential suitable habitat of *Nitraria tangutorum* during the current period (1970-2000).
